# Neuroantigen-Specific Autoregulatory CD8+ T Cells Inhibit Autoimmune Demyelination through Modulation of Dendritic Cell Function

**DOI:** 10.1371/journal.pone.0105763

**Published:** 2014-08-21

**Authors:** Venkatesh P. Kashi, Sterling B. Ortega, Nitin J. Karandikar

**Affiliations:** Department of Pathology, The University of Texas Southwestern Medical Center, Dallas, Texas, United States of America; Klinikum rechts der Isar der Technischen Universitaet Muenchen, Germany

## Abstract

Experimental autoimmune encephalomyelitis (EAE) is a well-established murine model of multiple sclerosis, an immune-mediated demyelinating disorder of the central nervous system (CNS). We have previously shown that CNS-specific CD8+ T cells (CNS-CD8+) ameliorate EAE, at least in part through modulation of CNS-specific CD4+ T cell responses. In this study, we show that CNS-CD8+ also modulate the function of CD11c+ dendritic cells (DC), but not other APCs such as CD11b+ monocytes or B220+ B cells. DC from mice receiving either myelin oligodendrocyte glycoprotein-specific CD8+ (MOG-CD8+) or proteolipid protein-specific CD8+ (PLP-CD8+) T cells were rendered inefficient in priming T cell responses from naïve CD4+ T cells (OT-II) or supporting recall responses from CNS-specific CD4+ T cells. CNS-CD8+ did not alter DC subset distribution or MHC class II and CD86 expression, suggesting that DC maturation was not affected. However, the cytokine profile of DC from CNS-CD8+ recipients showed lower IL-12 and higher IL-10 production. These functions were not modulated in the absence of immunization with CD8-cognate antigen, suggesting an antigen-specific mechanism likely requiring CNS-CD8-DC interaction. Interestingly, blockade of IL-10 *in vitro* rescued CD4+ proliferation and *in vivo* expression of IL-10 was necessary for the suppression of EAE by MOG-CD8+. These studies demonstrate a complex interplay between CNS-specific CD8+ T cells, DC and pathogenic CD4+ T cells, with important implications for therapeutic interventions in this disease.

## Introduction

Multiple sclerosis (MS) is an immune-mediated, demyelinating disorder of the central nervous system (CNS), believed to be mediated by autoreactive T cells. Studies in experimental autoimmune encephalomyelitis (EAE), a mouse model of MS, have established that myelin-reactive T cells contribute significantly to the pathology of MS. While the role of CD4+ T cells in immune pathogenesis and regulation is relatively well established, the role of CD8+ T cells remains poorly understood.

CD8+ T cells outnumber CD4+ T cells in human MS lesions and are oligoclonally expanded [1]–[5], indicative of an important function. Evidence exists for both pathogenic [6]–[13] and immune regulatory roles for CD8+ T cells in MS and EAE [12], [14]–[16]. For instance, human CD8+ T cells exhibit *in vitro* oligodendrocyte killing activity [17]. In EAE, myelin basic protein (MBP)-specific CD8+ T cells generated in the C3H background and myelin oligodendrocyte glycoprotein peptide (MOG_35–55_)-specific CD8+ T cells in C57BL/6 mice induce EAE [8], [9], [18], [19]. IL-17A secreting CD8+ T cells were recently shown to support MOG_37–50_-reactive Th17-mediated EAE [20]. In some transgenic models, CD8+ T cells were capable of pathogenic destruction in the CNS [18], [21]–[23]. In contrast, CD8−/− mice are known to develop more severe EAE as compared to wild-type mice [12], [15], [24] and lack of functional CD8+ T cells in β2-microglobulin deficient mice enhanced tissue damage in the CNS [16]. CD8+CD28- and CD8+CD122+ cells have been suggested to have a regulatory function [15], [25]. Using the WT-B6 model, we have recently shown that myelin oligodendrocyte glycoprotein (MOG_35–55_)-reactive CD8+ T (MOG-CD8+) cells are immune regulatory and can mitigate active and adoptive EAE [24], [26]. We have also shown that immune regulatory CNS-specific CD8+ T cells are present in clinically quiescent MS patients and in healthy individuals, and are uniquely deficient during clinical relapses of MS [27]. These clinically relevant findings underscore the importance of studying CNS-specific CD8+ T cells (CNS-CD8+) and their mechanisms of disease regulation.

Our previous studies identified the modulation of CD4+ T cells and antigen presenting cells (APCs) as possible mechanisms of disease suppression by CNS-CD8+. Professional APC subpopulations (dendritic cells, monocytes/macrophages and B cells) play important roles in not only T cell differentiation, but also in maintaining or modulating ongoing pathogenic T cell responses during disease [28]–[34]. In this study, we dissect the effects of CNS-CD8+ on the function of APC subsets, showing predominant modulation of CD11c+ dendritic cells (DC).

## Materials and Methods

### Mice

All mouse protocols were approved by the UT Southwestern Medical Center IACUC. C57BL/6 (B6) mice were purchased from UT Southwestern Medical Center mouse breeding core facility (Dallas, TX). IL-10 deficient mice were purchased from Jackson laboratory. OT-II mice were a kind gift from Dr. Chandrashekar Pasare. All mice were housed in UT Southwestern Animal Resource Center.

### EAE Induction and Evaluation

EAE in mice was induced as described previously [24], [26]. Briefly, 6–8 weeks-old female B6 mice were immunized subcutaneously in the flanks with 100 µg of MOG_35–55_ (MEVGWYRSPFSRVVHLYRNGK) or PLP_178–191_ (NTWTTCQSIAFPSK, UT Southwestern Protein Chemistry Technology Center) emulsified in complete Freund’s adjuvant (CFA) supplemented with 4 mg/ml *Mycobacterium tuberculosis* (MTB, H37Ra, Difco). On days 0 and 2 post-immunization, 250 ng of Pertussis toxin (PTX, List Biological Laboratories) was administered intraperitoneally in 100 µl of phosphate buffered saline (PBS). EAE severity was monitored daily and scored using the following scale: 0- no disease signs, 1- loss of tail tonicity, 2-hind limb weakness, 3- partial hind limb paralysis, 4- complete hind limb paralysis, 5- hind limb paralysis and forelimb weakness/moribund. Mice with grade 5 were sacrificed as per the protocol and counted as grade 5 for the reminder of the disease course.

### Adoptive Transfer of Antigen-Specific CD8+ T cells

CNS- and control (OVA)-CD8+ were generated as described previously [24], [26]. Briefly, splenocytes and lymph node cells were harvested at day 20 from mice immunized with 100 µg of either CNS- (MOG_35–55_ and PLP_178–191_) or control peptide (OVA_323–339_, ISQAVHAAHAEINEAGR) emulsified in CFA. Cells were cultured at 7×10^6^ cells/ml in the presence of 20 µg/ml of cognate peptide and 10 pg/ml of rIL-2. On day 3 of culture, live cells were isolated using Lympholyte-M (Cedarlane Laboratories, Burlington, NC) and CD8+ T cells were enriched by magnetic bead selection (Miltenyi Biotech, Germany). The purity of cells was typically >95%. 5×10^6^ CD8+ T cells were injected intravenously in 100 µl of PBS and the mice were immunized the following day with corresponding encephalitogenic peptide. EAE was monitored as mentioned above.

### CD4+ T cell Proliferation Assay

Splenic CD11c+, CD11b+ and B220+ cells were magnetically isolated, in that order, as per manufacturer’s instructions (Miltenyi Biotech, Germany) and used as antigen presenting cells (APC). CD4+ T cells from MOG_35–55_, PLP_178–191_ immunized (day 15–20) or naïve OT-II mice were used as responders. APCs (0.01×10^6^) and responders (0.2×10^6^) were co-cultured in a round-bottomed 96-well plate with or without 20 µg/ml of cognate antigen in a final volume of 200 µl. After 72 h in culture, cells were pulsed with 0.5 µCi/well of ^3^H-thymidine for 16 h. Cells were harvested on glass fiber mats and radioactivity was counted using Betaplate counter (Perkin Elmer, MA, USA). Background-subtracted counts per minute (ΔCPM) were used for proliferation analyses. For IL-10 inhibition assay, 4 µg/ml of anti-IL-10 antibody (eBioscience, clone JES5-2A5) or IgG1 isotype control was added to appropriate wells and the proliferation was measured as above.

### Cytokine Quantitation

IL-10, IL-12, IL-17 and IFN-γ were quantitated using antigen-capture ELISA as per manufacturer’s instructions (eBioscience, CA, USA). CD11c+, CD11b+ and B220+ cells were incubated at 1×10^6^/ml and stimulated with 250 ng/ml of lipopolysaccharide (LPS). Supernatants were harvested at various time points and stored at −20°C. For IFN-γ and IL-17, culture supernatants from replicates of CD4+ proliferation assays were harvested and stored at −20°C until use.

### Flow Cytometry

Anti-mouse CD11c-FITC, CD8-PE-Cy7, TCRβ-PE-Cy5.5, CD11b-Pacific Blue, B220-PE-Cy7, CD4-Pacific Blue, Foxp3-APC and CD86-PE antibodies were purchased from BD Biosciences. MHCII-AF700 and CD11c-Pacific Blue were purchased from Biolegend. 2×10^6^ cells were stained in PBS containing 5% fetal calf serum (FCS) and 0.1% w/v sodium azide at 4°C for 30 min, washed with the same buffer and fixed with 1% paraformaldehyde containing 2 mM EDTA. Data were acquired on 4-laser LSR II using FACSDiva software (Becton Dickinson) and analyzed using Flow Jo 9.0 software (Tree Star, OR). For splenic DC subset analyses, TCRβ- CD11c+ cells were gated on and the MHCII and CD86 expression levels evaluated in CD11c+CD8+ and CD11c+CD11b+ cells.

### Data Analysis

Statistical significance of differences in EAE scores, proliferation and cytokines were evaluated using two-tailed Student’s *t*-test. All statistical analyses were carried out using Graphpad Prism 6.0 software. Values of P≤0.05 were considered significant.

## Results

### Adoptive transfer of neuroantigen-specific CD8+ T cells inhibits APC function of DC, but not monocytes or B cells

**Figure 1:**
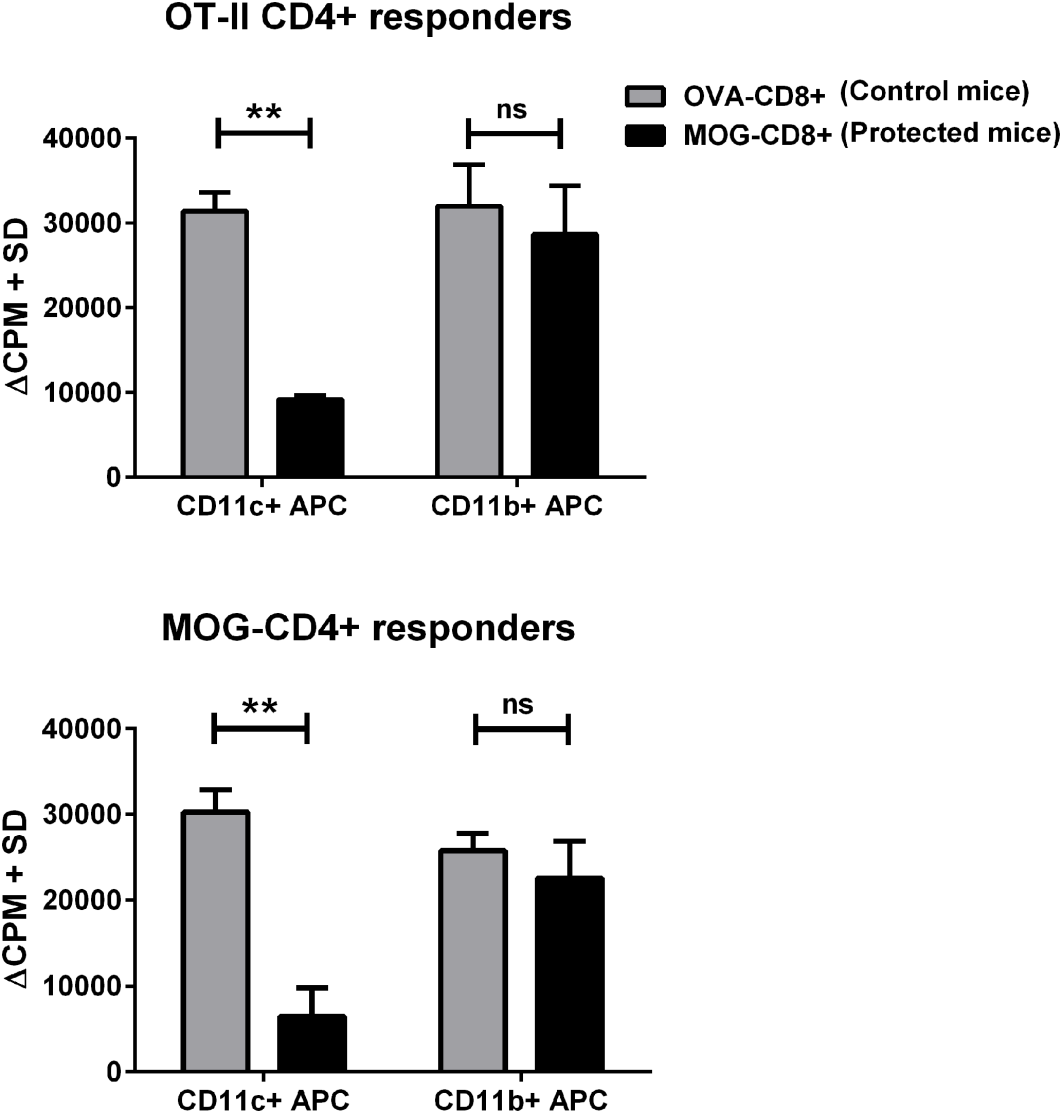
Adoptive transfer of neuroantigen-CD8+ T cells inhibits APC function of DC, but not monocytes. MOG-CD8+ (protected mice; black bars) and OVA-CD8+ (control mice; gray bars) T cells were transferred to naïve mice, followed by immunization with MOG_35–55_/CFA. Twelve days post-transfer, CD11c+ (DC) or CD11b+ (monocyte) populations were magnetically isolated from splenocyte preparations and cultured at 1∶20 ratio (APC:CD4) with CD4+ T cells derived from either naïve OT-II mice (top panel) or MOG_35–55_/CFA-immunized mice (MOG-CD4+, bottom panel), in the presence (or absence) of corresponding peptide antigens. Cultures were pulsed with ^3^H-thymidine on day 3 and harvested on day 4 for scintillation counting. Δ counts per minute (ΔCPM, background subtracted) are plotted on the y-axis. Data are representative of 3 independent experiments. (n = 15 per group). **p<0.01; ns = not significant.

We have previously shown that MOG-CD8+ T cells suppress EAE and that the mechanism of suppression, in part, involves modulation of APC function [26]. However, the specific APC targets of MOG-CD8+-mediated modulation are not known. In order to identify the APC subsets that may be affected, we adoptively transferred MOG-CD8+ or control OVA-CD8+ i.v. into naïve mice and induced active EAE with MOG_35–55_/CFA immunization. As previously observed [24], [26], MOG-CD8+ suppressed EAE significantly (Fig. S1, top panel) while the control OVA-CD8+ failed to modulate the disease. This was true even in the presence of OVA cognate antigen (Fig. S2). At various time points post-disease induction (days 7, 12, 30), we evaluated the antigen-presenting potential of magnetically sorted CD11c+ dendritic cells (DC), CD11b+ cells (APC: monocytes/macrophages), and B220+ cells (APC: B cells). Splenocytes-derived APC subsets were co-cultured with CD4+ T cells from either naïve OT-II TCR-transgenic mice or MOG_35–55_-immunized mice and proliferation to cognate antigen was measured. While DC from OVA-CD8+ recipient mice (non-protected controls) supported CD4+ T cell proliferation efficiently, DC from MOG-CD8+ recipients (protected mice) were significantly inefficient in activating OT-II CD4+ T cells as well as in reactivating MOG-CD4+ T cells (Fig. 1, p<0.01). This was true at all time points, including pre-disease onset and late in the disease course (Fig. S1, bottom panel). In contrast to DC, when CD11b+ monocytes/macrophages or B220+ B cells were used as APC, no significant differences were observed in activating OT-II CD4+ T cells or reactivating recall response from MOG-CD4+ T cells (Fig. 1 and Fig. S3A).

Next, we asked if the modulation of DC function was a property restricted to MOG_35–55_-specific CD8+ T cells or if this was also observable in CD8+ T cell specific to a different encephalitogenic peptide. Proteolipid protein derived peptide (PLP_178–191_)-specific CD8+ T cells (PLP-CD8+) were generated using the same method as that of MOG-CD8+ T cells. Similar to MOG-CD8+, PLP-CD8+ also suppressed active PLP_178–191_-induced EAE (Fig. S4, top panel). Importantly, DC from PLP-CD8+ recipient mice were also inefficient APC, confirming this functional modulation in the context of a different peptide (Fig. S4, bottom panel). Taken together, these data show that autoregulatory CNS-CD8+ specifically modulate DC function.

### DC subset distribution, viability, MHC class II and CD86 expression are not altered following CNS-CD8+ transfer

**Figure 2:**
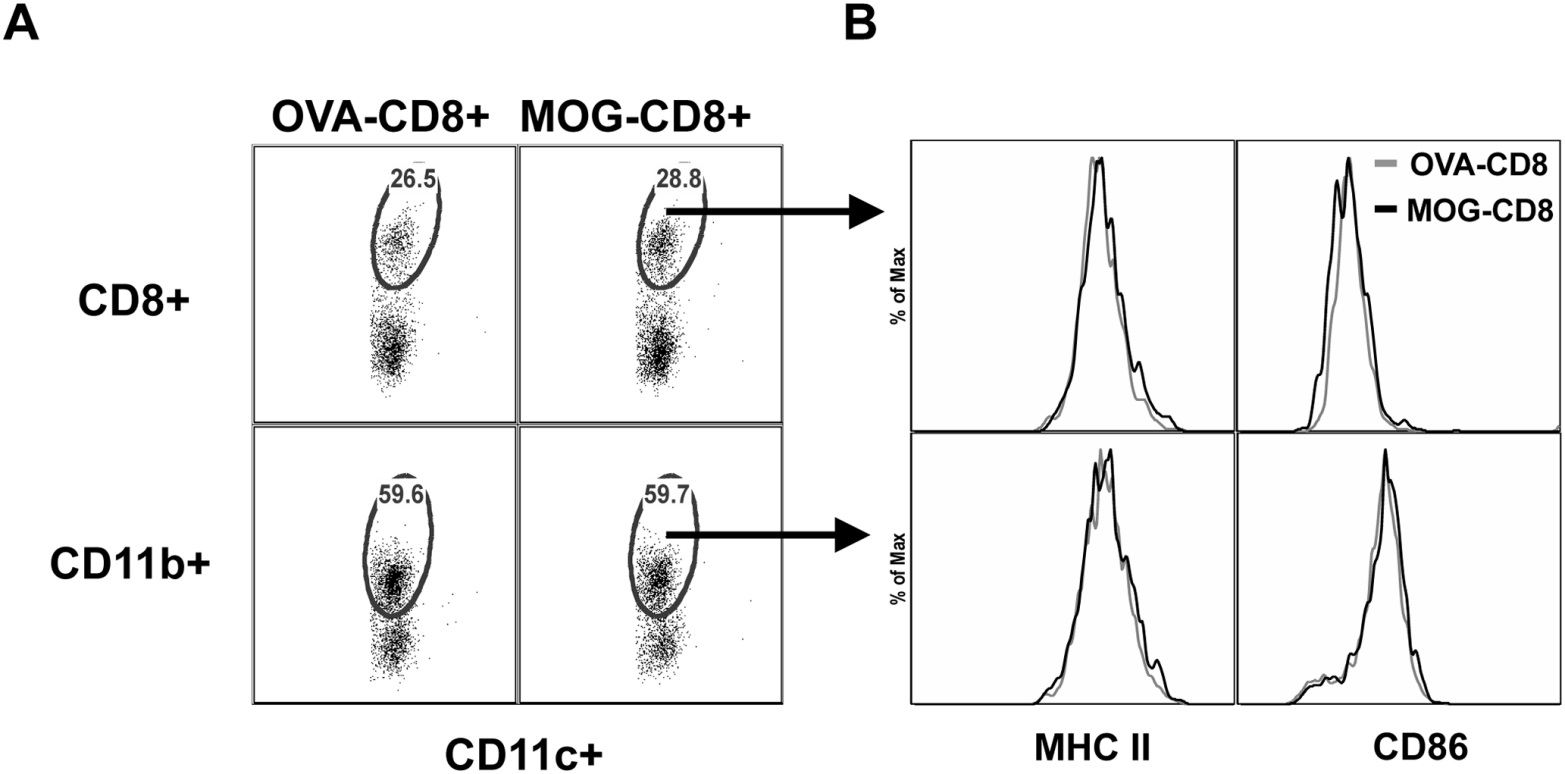
DC subset distribution, MHC class II and CD86 expression are not altered following MOG-CD8+ transfer. Splenocytes from MOG-CD8+ and OVA-CD8+ recipient mice were isolated and stained with fluorescently-labeled antibodies to evaluate: (**A**) percentage of DC subsets (CD8+CD11c+, top panel and CD11b+CD11c+, bottom panel) and (**B**) surface expression levels of MHC II and CD86. Representative data of 2 independent experiments (n = 8 per group).

Murine splenic DC are broadly classified into CD8+CD11c+ lymphoid and CD11b+CD11c+ myeloid DC. These subsets have been reported to play distinct roles in both immunity and in maintaining immune tolerance [35], [36]. Since we utilized positive selection of CD11c+ cells in the proliferation assays, it was possible that we sorted a heterogeneous population of DC and resulting APC function may be a byproduct of changes in subset distribution. In addition, the maturation status of the DC indicated by MHC class II and costimulatory molecule expression influences their ability to support CD4+ T cell proliferation. Therefore, to test for maturation status and subset variation, we enumerated the percent of CD11b+CD11c+ and CD8+CD11c+ DC subsets in protected and non-protected mice by flow cytometry and assessed MHC class II and CD86 expression. There were no significant changes in the percent of CD11b+CD11c+ or CD8+CD11c+ DC subsets observed between OVA- and MOG-CD8+ mice (Fig. 2A). Similarly, no differences in the expression of MHC Class II and CD86 were observed between OVA- and MOG-CD8+ mice as well as within the two DC subsets evaluated (Fig. 2B). In addition, expression levels of PD-L1 were not altered in the DC subsets between the groups (data not shown). Although there was a trend towards more DCs obtained from protected mice, the difference was not statistically significant when compared to the DC obtained from control mice (4×10^6^±1.05 vs. 2.03×10^6^±0.41, p = 0.1, n = 9). Finally, we did not observe significant difference between the two groups in the viability of the DCs obtained from the spleen (83.6±1.8 vs. 83.6±1.34, p>0.99, n = 8). These data suggest that the inefficient APC function is not due to inhibition of maturation, redistribution or viability of DC.

### CNS-CD8+ induce anti-inflammatory cytokine profiles of DC and CD4+ T cells

**Figure 3:**
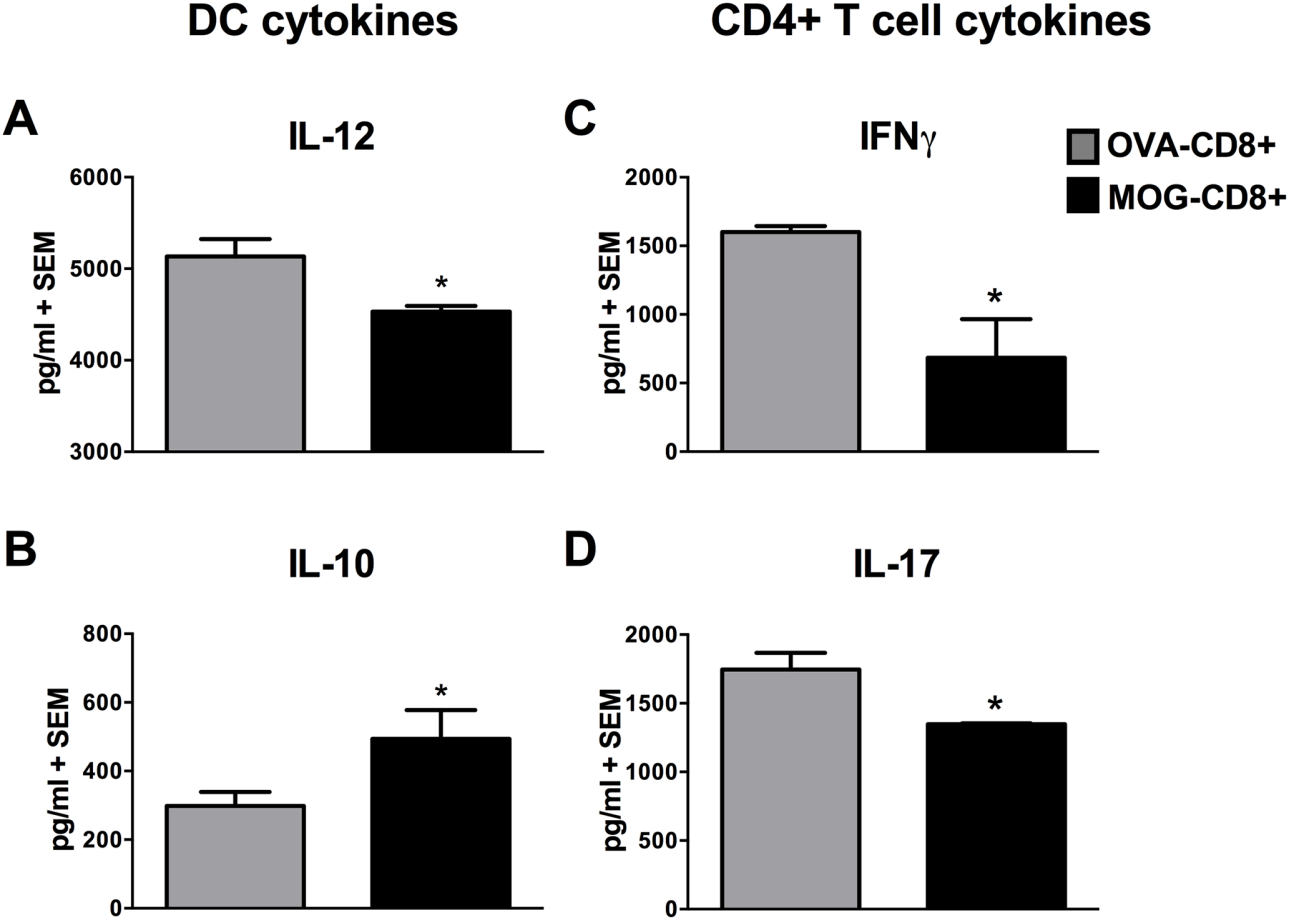
Cytokine profile of DC and MOG-CD4+ T cells. DC from protected and non-protected mice were magnetically isolated using CD11c+ beads and incubated at 1×10^6^/ml with 250 ng/ml of LPS. Culture supernatants were harvested and (**A**) IL-12 and (**B**) IL-10 was quantified using ELISA. In parallel experiments, DC were cultured with MOG-CD4+ T cells in the presence of 20 µg/ml of MOG_35–55_ and 72 h culture supernatants were harvested for (**C**) IFNγ and (**D**) IL-17 quantitation. Representative data from 2 independent (n = 10 per group) experiments are shown (*p<0.05).

Given the absence of differences in subset and MHC class II and CD86 expression between protected and non-protected mice, we explored the possibility of alterations in the cytokine profiles. To test this, DC were isolated from spleens as before and stimulated with LPS. Culture supernatants were harvested and secretion of IL-12 and IL-10 was evaluated by ELISA. Interestingly, while the levels of IL-12 secreted by DC from protected mice were significantly lower (4530±63.7 vs. 5131.5±192.4 pg/ml, p<0.05; Fig. 3A), the amount of IL-10 was significantly higher when compared to DC obtained from non-protected mice (493.3±84.4 vs. 298.13±41.15 pg/ml, p<0.05; Fig. 3B). Again, in contrast to DC, CD11b+ cells did not show significantly different IL-12 or IL-10 secretion between the two groups (Fig. S3B, S3C). B220+ cells from both groups did not secrete detectable amounts of IL-12 and produced similar amounts of IL-10 (Fig. S3B, S3C). Thus, DC from protected mice demonstrate an anti-inflammatory cytokine profile.

Cytokines secreted by APC play an important role in governing the activation and differentiation of CD4+ T cells in autoimmune disorders [37]. Since DC demonstrated an anti-inflammatory cytokine profile, we next evaluated the cytokine profile of CD4+ T cells stimulated by these DC. For this, culture supernatants were harvested from replicate proliferation CD4 proliferation assays. Correlating with the decreased overall proliferation of CD4 T cells (Fig. 1), we also observed a reduction in pro-inflammatory IFN-γ (1333.7±438.2 vs. 1622.6±104.8 pg/ml, Fig. 3C, p<0.05) and IL-17 (1346.7±6.85 vs. 1745.4±122.6 pg/ml, Fig. 3D, p<0.05) secretion when DC from protected mice were used as APC. Overall, these data suggest that DC from protected mice induce reduced proliferative and cytokine responses from CD4 T cells.

In some studies, IL-10-producing DC have been reported to generate induced CD4+FOXP3+ Tregs [38]. Therefore, we also evaluated whether CNS-CD8+ treatment resulted in modulation of Treg numbers. While Treg numbers were equivalent in protected vs. control mice early in the disease course, there were significantly elevated numbers of CD4+FOXP3+ Tregs in CNS-CD8+ protected mice on days 13 and 20 post-CD8 transfer (Fig. S5).

### Immunization with cognate antigen is required for DC modulation

**Figure 4:**
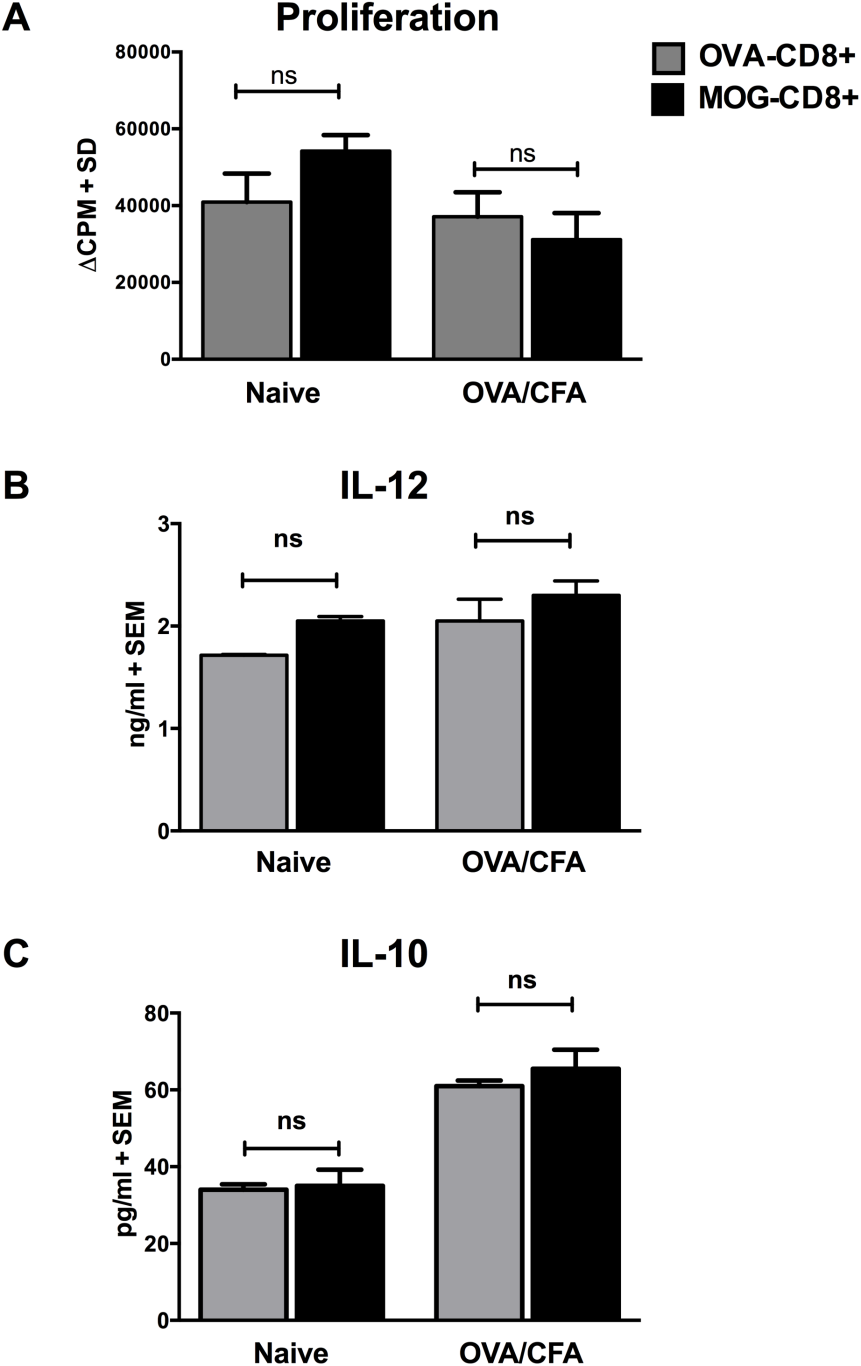
Immunization with cognate antigen is required for DC modulation. MOG-CD8+ or OVA-CD8+ T cells were transferred intravenously into naïve mice, followed by either no immunization or OVA/CFA immunization. Seven days post transfer, CD11c+ DC were isolated from spleen and were either (**A**) used as APCs in ^3^H-thymidine-based proliferation assay with MOG-CD4+ T cells as responders (y-axis corresponds to ΔCPM), or stimulated at 1×10^6^/ml with 250 ng/ml of LPS, followed by measurement of (**B**) IL-12 and (**C**) IL-10 in the supernatants. Representative data of 2 independent experiments are shown (n = 6 per group). ns = not significant.

Recent studies suggested that CD8+ T cells can modulate bone marrow-derived DC in an antigen-independent manner *in* *vitro* [39]. Given that MOG-CD8+ T cells modulate the function of DC in active EAE, we wanted to know if this was an antigen-specific phenotype. To address this question *in* *vivo*, OVA- and MOG-CD8+ T cells were transferred to naïve mice and the function of DC was evaluated in the absence of EAE induction by MOG_35–55_. Seven days post-CD8+ T cell transfer, splenic DC were isolated and used as APCs in a MOG-CD4+ recall response. Additionally, a group of CD8+ T cell recipient mice were immunized with OVA_323–339_/CFA and the function of DC was evaluated as before. Transfer of CNS-CD8+ T cells into naïve or OVA_323–339_/CFA immunized mice did not significantly modulate the antigen-presenting potential (Fig. 4A) or cytokine profile (Fig. 4B) of DC, suggesting that cognate antigen presentation was required for CNS-CD8+ T cells to influence DC activity.

### DC-derived IL-10 is required for EAE suppression by MOG-CD8+ T cells

**Figure 5:**
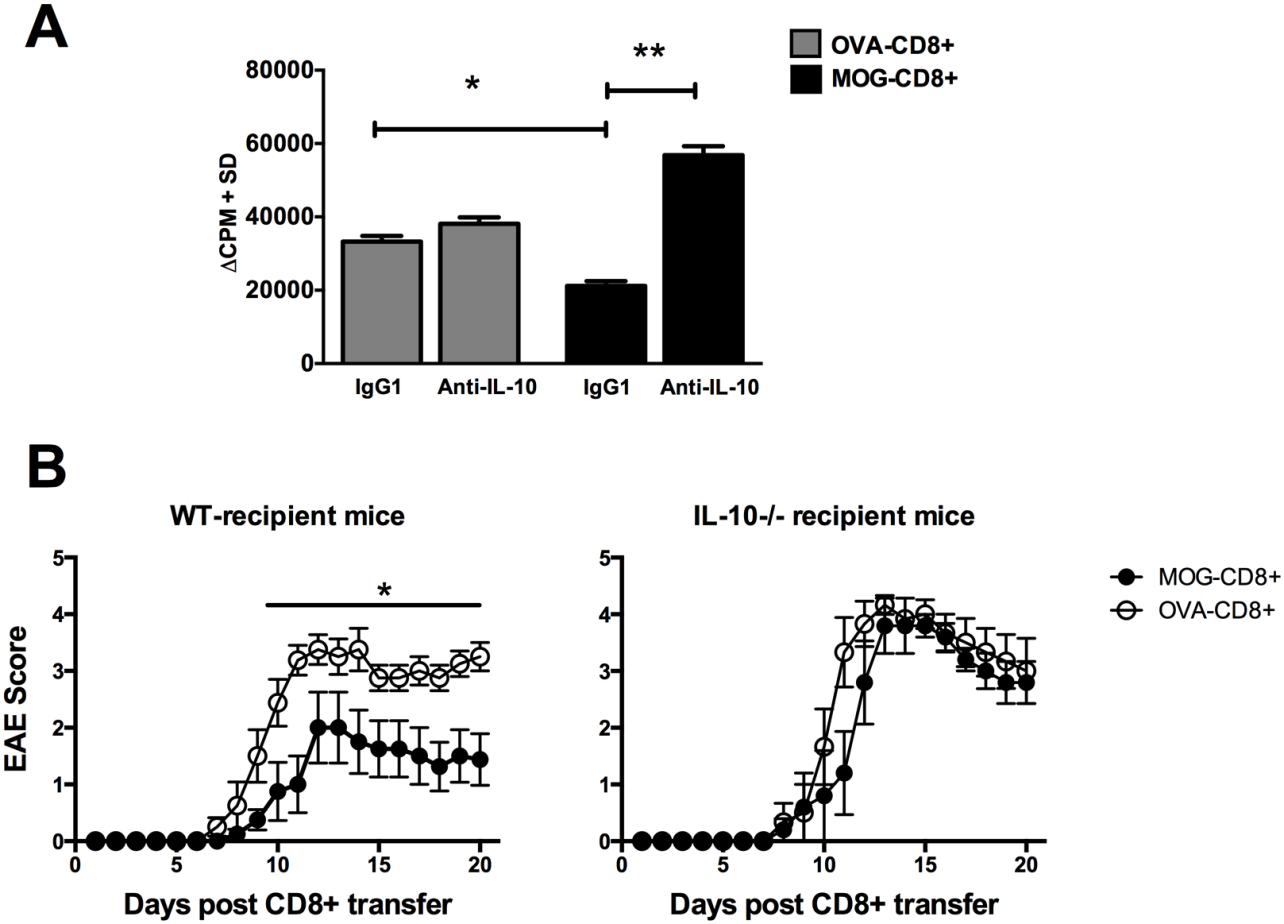
DC-derived IL-10 is required for modulation of EAE by MOG-CD8+ T cells. (**A**) Effect of DC-derived IL-10 on CD4+ T cell proliferation was evaluated using a thymidine-incorporation assay. Magnetically sorted DC from OVA-CD8+ or MOG-CD8+ recipient mice were co-cultured with CD4+ T cells from MOG_35–55_/CFA immunized mice. 4 µg/ml of anti-IL-10 antibody or IgG isotype control was added to the indicated cultures. (**B**) WT MOG-CD8+ and OVA-CD8+ T cells were transferred to either naïve wild-type (left panel) or IL-10−/− (right panel) mice, followed by EAE induction. Mean EAE scores are plotted on the y-axis vs. days post-transfer on the x-axis. Data represent two independent experiments, with 6–8 mice per group (*p<0.05).

IL-10 is an anti-inflammatory cytokine and contributes significantly in the suppression of autoimmune diseases. The observation that DC from protected mice were inefficient APC and secreted higher amounts of IL-10 raised the possibility that inhibition of CD4+ T cell proliferation was IL-10 dependent. Therefore, we repeated proliferation assays using DC derived from protected mice and MOG-CD4+ as responders in the presence of an IL-10 blocking antibody or isotype control. While DC from protected mice were inefficient APC in neutral cultures, blockade of IL-10 resulted in a significant increase in CD4+ T cell proliferation (Fig. 5A; p<0.01). Cultures with DC from non-protected mice, showed a modest increase in the proliferation of CD4+ T cells in the presence of anti-IL-10. These data suggest that IL-10 secreted by the DC inhibits the proliferation of auto-reactive CD4+ T cells, which may connect the DC phenotype to suppression of EAE.

Thus, we finally tested if IL-10 expression was required *in* *vivo* for MOG-CD8+ T cells to ameliorate EAE severity. WT MOG- or OVA-CD8+ were transferred to wild-type (WT) or IL-10 deficient (IL-10−/−) mice and active EAE was induced by MOG_35–55_/CFA immunization. As expected, MOG-CD8+ T cells attenuated the EAE symptoms in WT-recipient mice (mean maximum scores of 2±1.7 vs. 3.9±0.8 (p<0.05) (Fig. 5B, left panel). Interestingly, the EAE-suppressive function of MOG-CD8+ T cells was absent when they were transferred to IL-10−/− recipient mice (mean maximum scores of 4.2±0.4 vs. 4.3±0.5) (Fig. 5B, right panel). Taken together, these data are consistent with a model where MOG-CD8+ T cells exert their disease regulatory function by modulating DC to secrete IL-10, which in turn attenuates pathogenic CD4 responses and EAE.

## Discussion

The important role of CD4+ T cells in EAE pathogenesis and regulation has been long established and extensively studied. In contrast, the role of CD8+ T cells, particularly CNS-specific CD8+ cells, is poorly understood. It is now known that CD8+ T cells out number CD4+ cells in the human MS plaques [1], [2] and undergo oligoclonal expansion at the site of pathology [3]–[5], indicating an important functional role at the site of pathology. Given that CD8+ T cells are generally associated with cytotoxic killing of target cells, it is logical to predict that CNS-specific CD8+ T cells should have a pathogenic function. *In vitro* studies have shown cytotoxicity of human CD8+ T cells toward oligodendrocytes [17]. Certain EAE studies also suggest a pathogenic role, such as MBP-specific CD8+ T cells in C3H mouse [9], [10] and MOG_35–55_-specific CD8+ T cells in B6 mouse [8], [11]. Additionally, mouse models based on the use of TCR-transgenic CD8+ T cells and sequestered expression of heterologous antigen in the CNS or HLA-transgenic mice also suggest a potential pathogenic function [21], [40], [41]. In contrast, studies have also shown a regulatory role for CD8+ T cells, both in EAE and MS [12], [14]–[16], [24], [26], [27]. The paucity in our understanding of the role of CNS-specific CD8+ T cells underscores the need for further studies and mechanistic dissection.

Using the WT B6 model, we have shown that MOG-specific CD8+ T cells suppress EAE severity [26], while the OVA-CD8+ lack this immune suppressive property. One possible explanation for this observation could be that auto-antigen specific CD8+ T cells may harbor a regulatory population that may be absent in the CD8+ T cells generated against foreign antigens like OVA. Indeed, in other autoimmune disease models, low avidity CD8+ regulatory T cells have been described [42]. Mechanistically, we have shown that CNS-CD8+ T cells may target the autoreactive CD4+ T cells [24] as well as the APC [26]. However, the APC subsets targeted by the CD8+ T cells were not known and this was the focus of the current study. We now show that the predominant effects of CNS-CD8+ are exerted on CD11c+ DC, with no detectable changes observed in either CD11b+ monocytes/macrophages or B220+ B cells. This is in contrast to certain other EAE modulating agents, which work through induction of Type II monocytes/macrophages [31], [43] or modulation of B cell function [32], [44]. CNS-CD8+ transfer results in DCs that are inefficient in activating naïve OT-II CD4+ cells and in supporting recall responses from CNS-CD4+ T cells. DC functional changes were observed fairly early in the disease course (prior to disease onset) and lasted long term. Thus, these DC are likely unable to maintain ongoing CD4+ T cell responses *in vivo* and certainly deficient in priming new pathogenic responses from naïve T cells (such as those required for epitope spreading) [45], [46]. Our observation that B cell functions were not affected by MOG-specific CD8+ T cells may be based on the specific model used in our experiments, in that the MOG_35–55_/CFA-induced model of EAE does not require the antigen presenting function of B-cells. It is possible that in a B-cell-dependent model of EAE, such as that induced with recombinant MOG protein, one may see effects of CD8-mediated autoregulation on B cells and this remains to be explored. Monocytes/macrophages on the other hand can cross-present CD8+ epitopes in this model and therefore it is intriguing that these APCs were not modulated. A possible explanation for this phenotype could be that DCs are more efficient in cross-presentation [47],[48] and given that CNS-CD8+ are of low avidity, robust antigen cross-presentation may be essential for the regulatory CD8+ T cells to target the APC.

The inefficiency in DC function was not due to changes in their maturation status or subset distribution. Given that DC play important roles in both immune activation and regulation [49]–[52], altering their phenotype towards a non-inflammatory phenotype can have a dampening effect on autoimmunity. As expected, IFN-γ and IL-17, two inflammatory cytokines implicated in EAE pathology, were secreted at lower levels by MOG-specific CD4+ T cells when DC from protected mice were used as APCs. This may, of course, be a simple reflection of lower proliferation of these cells in culture. In previous studies, we had observed that bulk APC from protected mice were inefficient at stimulating CD4 proliferation, despite using antigen-independent mitogenic stimulation with Con A [26]. Since maturation status of DC was not the cause for the inhibition of CD4+ T cells, we hypothesized that a soluble factor secreted by the DC actively inhibits CD4 proliferation. We thus tested the cytokine profile of DC following LPS stimulation and found that DC secreted significantly lower levels of total IL-12 but higher amounts of IL-10. It is unclear whether fewer inflammatory DC are present in the protected mice or if they are hyporesponsive to LPS. The likely mechanism by which CNS-CD8+ T cells modulate DC function can be determined by the effector molecules expressed by these cells. In line with this, we have recently demonstrated that MOG-CD8+ T cells express perforin and IFN-γ both of which are needed to suppress EAE, while IL-4 and IL-10 are not essential [24]. Also, MOG-CD8+ T cells reduce inflammatory Th1/Th17 CD4+ T cells in the CNS as well as peripheral lymphoid organs corroborating the cytotoxic potential of CNS-CD8+ [24]. Therefore, it is also possible that MOG-CD8+ T cells cytotoxically eliminate pro-inflammatory DC. Our preliminary intracellular cytokine staining data seem to suggest that there are fewer IL-12-producing cells in protected mice (data not shown). Recent studies in EAE have also shown that CD11b+ DC in the CNS cross-present myelin antigens to CD8+ T cells and also suggest that these DC can be eliminated by myelin-reactive CD8+ T cells [46]. Alternatively, MOG-CD8+ may modulate DC differentiation toward an anti-inflammatory or tolerogenic phenotype, indirectly through IFN-γ-mediated IDO (indoleamine-2, 3-deoxygenase) induction, as seen in other autoimmune disease settings [42]. We have also shown that adoptively transferred MOG-CD8+ T cells traffic to the CNS of mice immunized with MOG_35–55_/CFA [24]. It is possible that MOG-CD8+ T cells modulate/kill DC in the CNS as a part of their immunomodulatory function and this is an active focus of our ongoing investigation. Taken together, the regulatory activity of CNS-CD8+ T cells involves IFN-γ-mediated modulation as well as perforin-mediated cytotoxic elimination of pro-inflammatory cells [53].

IL-10 is an anti-inflammatory cytokine with paracrine and autocrine effects [54]. Interestingly, the autocrine effects of IL-10 on DC include decrease in the ability to secrete IL-12 with no significant changes in the MHC class II and CD86 expression [55], a phenotype that is consistent with our observations. We thus tested whether IL-10 was the effector molecule from DC that actively inhibited CD4+ T cell proliferation, by using anti-IL-10-mediated blockade. While we saw a modest increase in the proliferation of CD4+ T cells incubated with DC from non-protected mice, CD4+ T cells incubated with DC from protected mice showed a significant (>2 fold) increase in proliferation in the presence of anti-IL-10. These data confirmed that IL-10 from DC in MOG-CD8+ T cell mice actively inhibited the proliferation of CD4+ T cells, probably resulting in a decrease in IFN-γ and IL-17 secretion. Furthermore, IL-10 producing splenic DC have been reported to generate induced CD4+ Tregs [56]. Along those lines, we observed an increased number of splenic CD4+Foxp3+ cells in CNS-CD8+-protected mice and this is correlated with the presence of IL-10 producing DC in the spleen. This is seen in the context overall reduced DC-supported proliferation of CD4+ T cells in vitro with reduced Th1/Th17 differentiation. This raises the possibility that DC in protected mice may help skew the CD4 response toward a regulatory phenotype with a reduction in proliferative potential. Whether these CD4+ Tregs are MOG-specific and contribute to disease suppression requires further investigation.

Finally, we tested the relevance of IL-10 *in vivo* using IL-10−/− mice. In previous studies we have shown that CD8-instrinsic IL-10 was not required for their suppressive function, i.e., MOG-CD8+ derived from IL-10−/− mice were capable of inhibiting EAE in WT mice [24]. In the current studies, we asked the reverse question by testing whether WT MOG-CD8+ could inhibit disease in an IL-10-deficient setting. While MOG-CD8+ T cells showed the expected suppression of EAE in WT-mice, suppression was lost when these cells were transferred to IL-10−/− mice. Since the IL-10 was not specifically deficient in just the DC subset, it is possible that more global effects of IL-10, such as neuroprotection [57], [58] contributed to our *in vivo* findings. Future studies will be needed to dissect these possibilities *in vivo*. Overall, our results show that MOG-CD8+ T cells suppress EAE in an IL-10-dependent manner and are consistent with the model where DC-derived IL-10 is important to mediate this suppression.

In this study, we demonstrate that neuroantigen-specific CD8+ T cells suppress EAE by modulating DC function in a cognate antigen-dependent fashion, with consequent inhibition of pathogenic CD4+ T cells. This mechanism of peripheral and CNS immune modulation may prove to be an attractive avenue for therapeutic intervention in autoimmune disease settings.

## Supporting Information

Figure S1 **Kinetic analysis of DC modulation.** Top panel represents typical suppression of EAE by MOG-CD8+ T cells. Closed circles correspond to MOG-CD8+ and open circles to OVA-CD8+ recipients. Time points tested have been highlighted by open ovals. DC from day 7 and day 30 post-CD8+ T cell transfer were isolated and used as APC in ^3^H-Thymidine incorporation assay (ΔCPM shown on y-axis, bottom panel). Data are representative of at least 2 independent experiments (n = 10 per group). *p<0.05.
(TIF)

Figure S2 **OVA-CD8+ do not modulate EAE severity.** Lymph node and spleen cells from MOG_35–55_, PLP-_178–191_ and OVA_323–339_ immunized mice were cultured in the presence of cognate antigen for 3 days. CD8+ T cells were magnetically sorted and injected into recipient B6 mice i.v. Mice were immunized with MOG-OVA peptide (MEVGWYRSPFSRVVHLYRNGK-ISQAVHAAHAEINEAGR, which elicits EAE symptoms similar to MOG_35–55_/CFA). Pertussis toxin was injected on day 0 and 2 and EAE severity was evaluated daily. In the absence of PLP_178–191_/CFA-immunization in the recipient mice, PLP-CD8+ do not suppress EAE and hence serve as negative control. Representative data from 2 independent experiments are shown (n = 10 per group). Ns = not significant *p<0.05.
(TIF)

Figure S3 **CD11b+ and B220+ cells are not modulated by MOG-CD8+ T cells.** CD11b+ and B220+ cells magnetically sorted from OVA-CD8+ or MOG-CD8+ recipient mice were either **(A)** used as APC in thymidine-incorporation assays using MOG-specific CD4+ T cells as responders (ΔCPM shown) or stimulated with LPS at 1×10^6^/ml cells, followed by measurement of culture supernatants for **(B)** IL-12 and **(C)** IL-10. ns = not significant; nd = not detected.
(TIFF)

Figure S4 **Transfer of PLP_178–191_ CD8+ T cells modulates DC function.** Upper panel represents typical EAE disease pattern induced by PLP_178–191_/CFA immunization and its suppression by PLP-CD8+ T cells. Closed circles correspond to PLP-CD8+ and open circles to OVA-CD8+ recipients. Lower panel shows assessment of DC for APC function using thymidine-incorporation assays (ΔCPM plotted on the y-axis). Data are representative of at least 2 independent experiments (*p<0.05).
(TIFF)

Figure S5 **CNS-CD8+ recipient mice have increased CD4+Foxp3+ cells.** Splenocytes from control- and CNS-CD8 recipient mice isolated on days 7, 13 and 20 post-CD8+ transfer were stained with fluorescently tagged antibodies and the percent TCRvβ+CD4+Foxp3+ cells quantitated by flow cytometry. Representative data of 2 or more independent experiments are shown (n = 10 per group). *p<0.05, ***p<0.001, ns = not significant.
(TIFF)
