# Supplementary figures and images for: Neuroantigen-Specific Autoregulatory CD8+ T Cells Inhibit Autoimmune Demyelination through Modulation of Dendritic Cell Function
Source: PLoS One. 2014 Aug 21;9(8):e105763. doi: 10.1371/journal.pone.0105763 (PMC4140828; doi:10.1371/journal.pone.0105763)

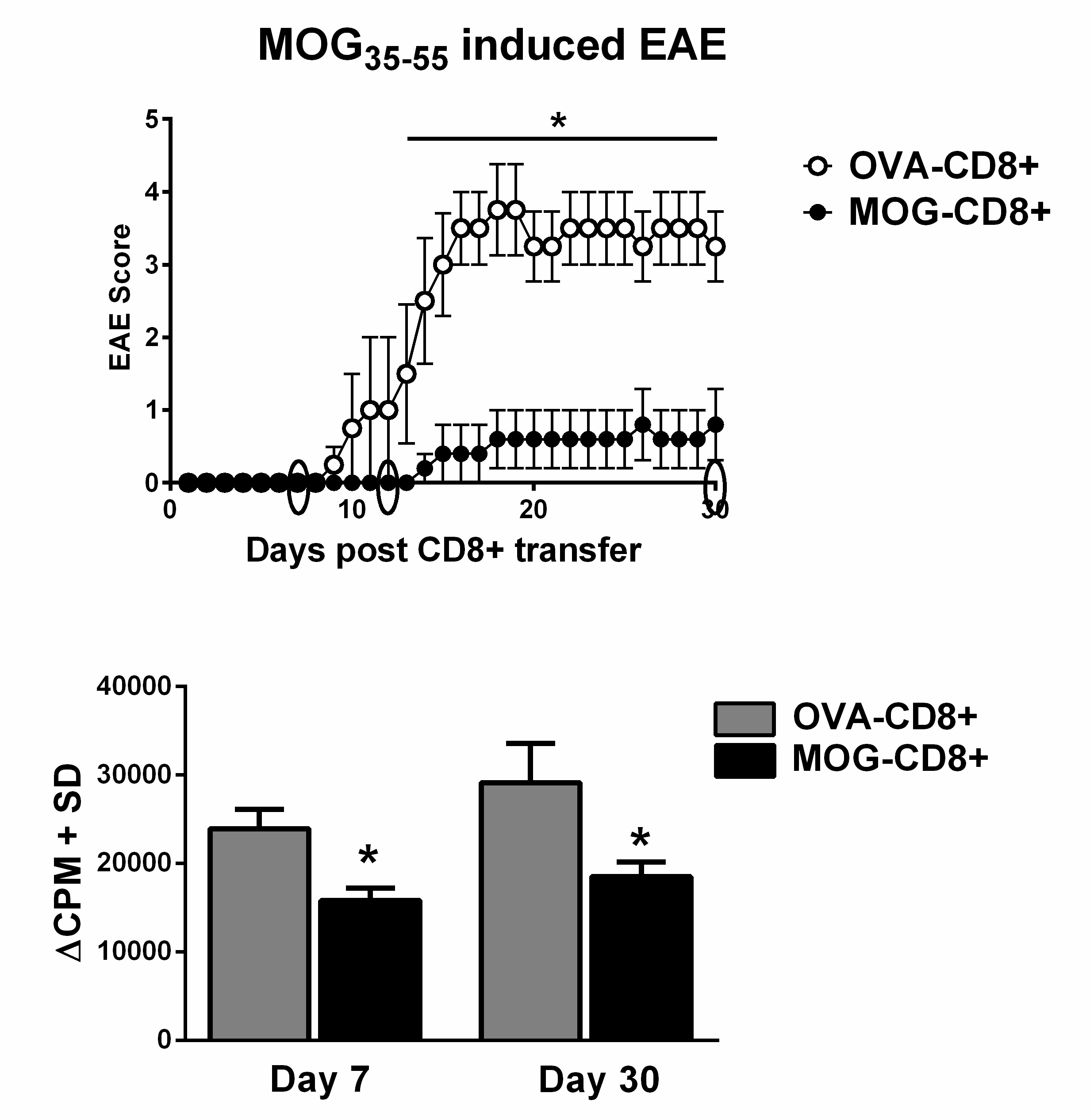

Supplement: Figure S1 — Kinetic analysis of DC modulation. Top panel represents typical suppression of EAE by MOG-CD8+ T cells. Closed circles correspond to MOG-CD8+ and open circles to OVA-CD8+ recipients. Time points tested have been highlighted by open ovals. DC from day 7 and day 30 post-CD8+ T cell transfer were isolated and used as APC in 3H-Thymidine incorporation assay (ΔCPM shown on y-axis, bottom panel). Data are representative of at least 2 independent experiments (n = 10 per group). *p<0.05. (TIF) [file pone.0105763.s001.tif]

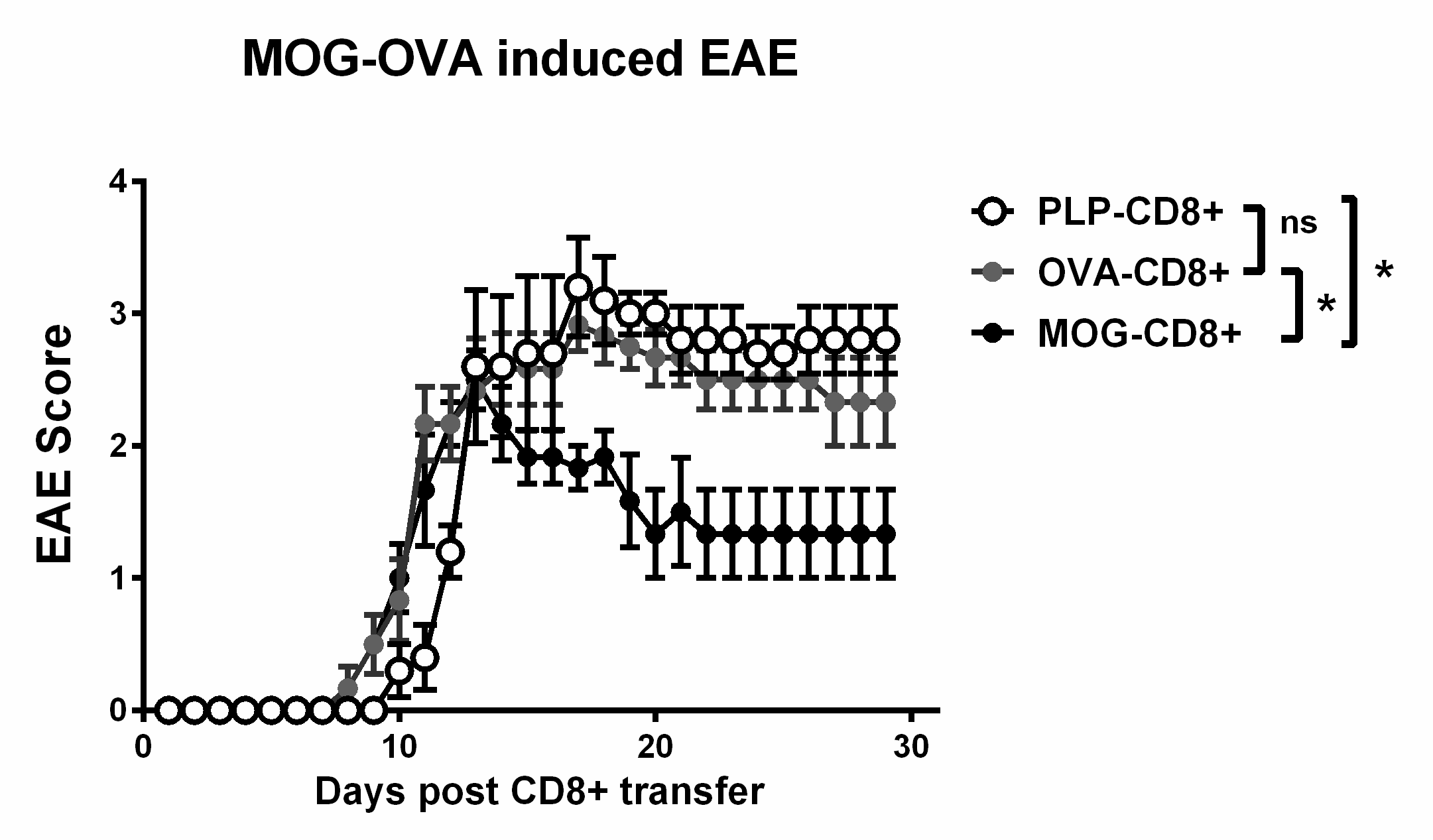

Supplement: Figure S2 — OVA-CD8+ do not modulate EAE severity. Lymph node and spleen cells from MOG35–55, PLP-178–191 and OVA323–339 immunized mice were cultured in the presence of cognate antigen for 3 days. CD8+ T cells were magnetically sorted and injected into recipient B6 mice i.v. Mice were immunized with MOG-OVA peptide (MEVGWYRSPFSRVVHLYRNGK-ISQAVHAAHAEINEAGR, which elicits EAE symptoms similar to MOG35–55/CFA). Pertussis toxin was injected on day 0 and 2 and EAE severity was evaluated daily. In the absence of PLP178–191/CFA-immunization in the recipient mice, PLP-CD8+ do not suppress EAE and hence serve as negative control. Representative data from 2 independent experiments are shown (n = 10 per group). Ns = not significant *p<0.05. (TIF) [file pone.0105763.s002.tif]

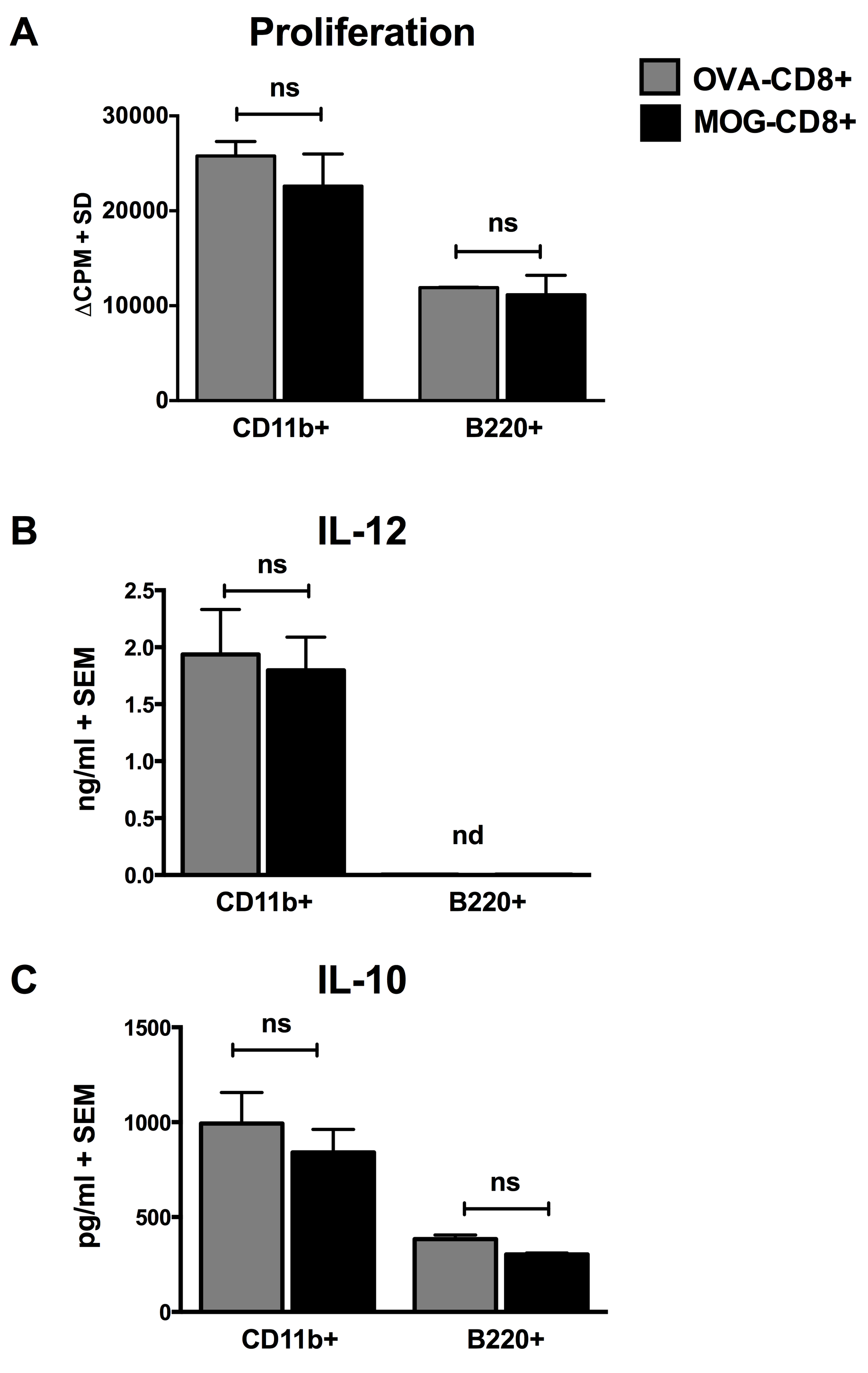

Supplement: Figure S3 — CD11b+ and B220+ cells are not modulated by MOG-CD8+ T cells. CD11b+ and B220+ cells magnetically sorted from OVA-CD8+ or MOG-CD8+ recipient mice were either (A) used as APC in thymidine-incorporation assays using MOG-specific CD4+ T cells as responders (ΔCPM shown) or stimulated with LPS at 1×10^6^/ml cells, followed by measurement of culture supernatants for (B) IL-12 and (C) IL-10. ns = not significant; nd = not detected. (TIFF) [file pone.0105763.s003.tiff]

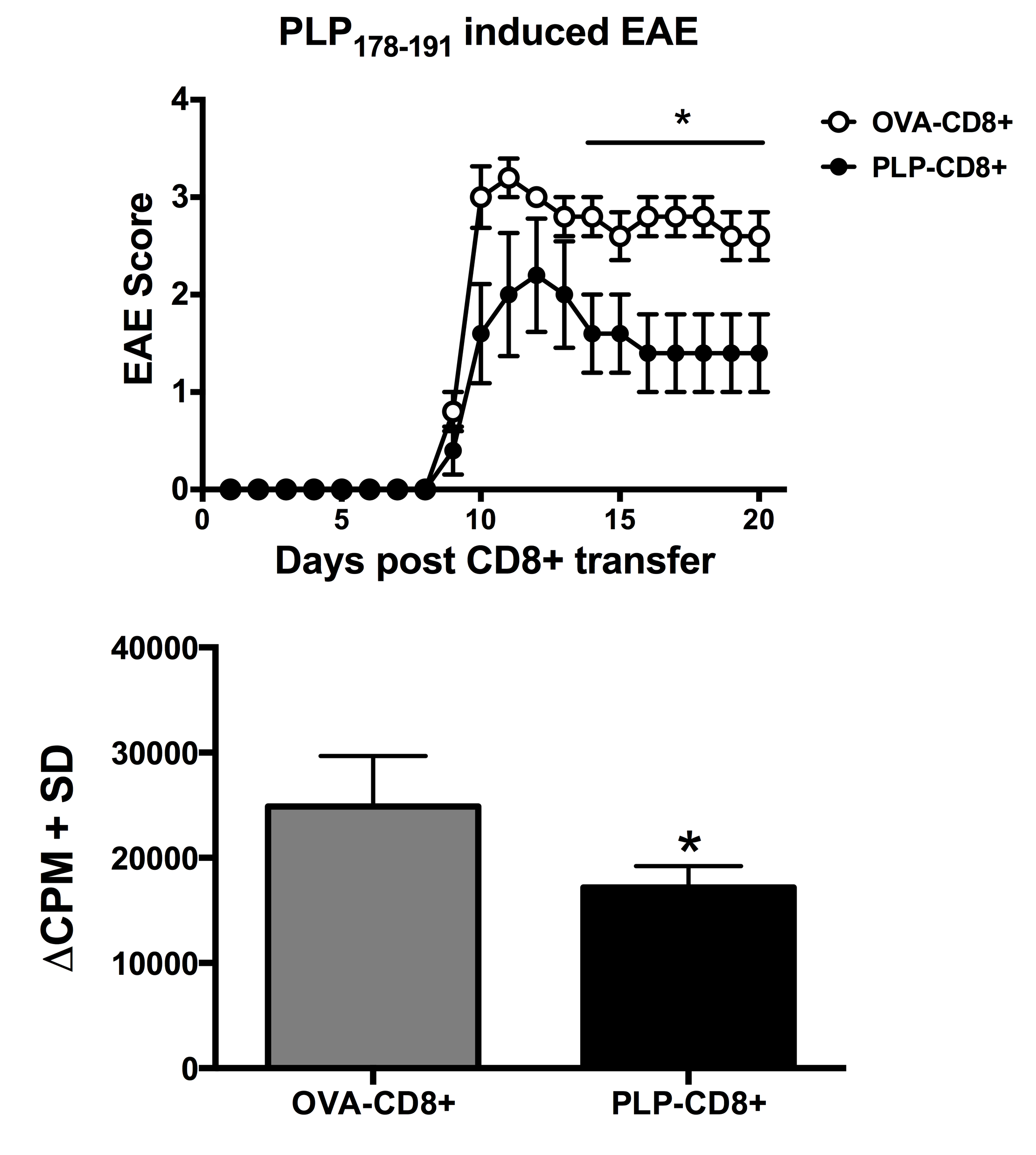

Supplement: Figure S4 — Transfer of PLP178–191 CD8+ T cells modulates DC function. Upper panel represents typical EAE disease pattern induced by PLP178–191/CFA immunization and its suppression by PLP-CD8+ T cells. Closed circles correspond to PLP-CD8+ and open circles to OVA-CD8+ recipients. Lower panel shows assessment of DC for APC function using thymidine-incorporation assays (ΔCPM plotted on the y-axis). Data are representative of at least 2 independent experiments (*p<0.05). (TIFF) [file pone.0105763.s004.tiff]

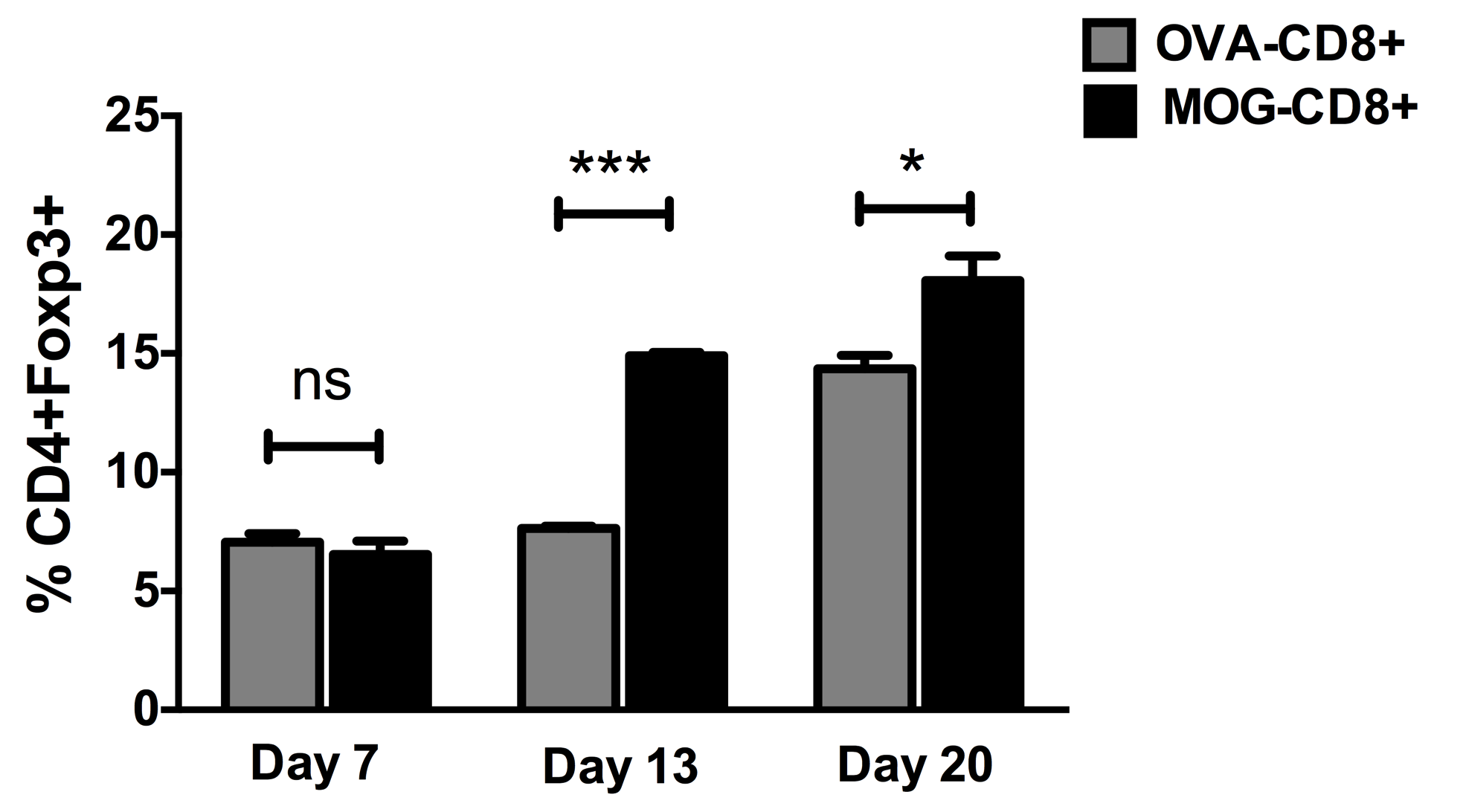

Supplement: Figure S5 — CNS-CD8+ recipient mice have increased CD4+Foxp3+ cells. Splenocytes from control- and CNS-CD8 recipient mice isolated on days 7, 13 and 20 post-CD8+ transfer were stained with fluorescently tagged antibodies and the percent TCRvβ+CD4+Foxp3+ cells quantitated by flow cytometry. Representative data of 2 or more independent experiments are shown (n = 10 per group). *p<0.05, ***p<0.001, ns = not significant. (TIFF) [file pone.0105763.s005.tiff]
